# Molecular Characterization of Two Toll Receptors in *Hyriopsis cumingii* and Their Potential Roles in Antibacterial Response

**DOI:** 10.3389/fphys.2019.00952

**Published:** 2019-07-25

**Authors:** Ying Huang, Guosong Zhang, Qian Ren

**Affiliations:** ^1^College of Oceanography, Hohai University, Nanjing, China; ^2^School of Agriculture and Bioengineering, Heze University, Heze, China; ^3^Co-Innovation Center for Marine Bio-Industry Technology of Jiangsu Province, Lianyungang, China; ^4^College of Marine Science and Engineering, Nanjing Normal University, Nanjing, China

**Keywords:** *Hyriopsis cumingii*, innate immunity, Toll receptors, RNAi, antibacterial response

## Abstract

Tolls/Toll-like receptors (TLRs) play a key role in innate immunity by detecting the invading microbes and subsequently activating downstream signaling cascades. In this study, two new molluscan Toll members (designed as *HcToll6* and *HcToll7*) were identified from triangle-shell pearl mussel (*Hyriopsis cumingii*). The obtained *HcToll6* full-length cDNA was 3207 bp consisting of a 2223 bp open reading frame (ORF) that encoded a peptide of 740 amino acids. *HcToll7* cDNA is a 3216 bp molecule that contains an ORF of 2139 bp encoding a protein of 712 amino acids. The deduced *HcToll6* and *HcToll7* proteins share two common structures: extracellular leucine-rich repeat (LRR) domains and intracellular Toll/interleukin-1 receptor (TIR) domain. Quantitative real-time PCR results showed that *HcToll6* and *HcToll7* were mainly expressed in the hepatopancreas and the gills, and they responded rapidly to bacterial stimulation. RNA interference by dsRNA results revealed that *HcToll6* and *HcToll7* RNAi strongly decreased the expression of lysozyme (*HcLyso*) and defensin (*HcDef*) in the gills of RNAi-treated mussels with *Vibrio parahaemolyticus* challenge. As a pattern recognition receptor, the prokaryotic expressed the recombinant LRR domains of *HcToll6* and *HcToll7* (rHcToll6-LRR and rHcToll7-LRR) could bind to Gram-positive and Gram-negative bacteria and had a strong tendency to recognize lipopolysaccharide (LPS) and peptidoglycan (PNG). rHcToll6-LRR and rHcToll7-LRR exhibited a significant *in vitro* bactericidal activity against *V. parahaemolyticus* and *Staphylococcus aureus*. These findings provide useful information to characterize Tolls in mussels.

## Introduction

Lacking a true adaptive immune system, invertebrates against infectious agents mainly rely on innate immunity (Loker et al., 2004). Innate immune response is the main line of defense, triggering various humoral and cellular activities through signal transduction pathways to defense against microbial infections (Akira et al., 2006). Toll, immune deficiency (IMD), and Janus family tyrosine kinase and signal transducer and activator of transcription (JAK/STAT) pathways are regarded as the three main pathways regulating the immune response of invertebrates (Li and Xiang, 2013). During pathogen invasion, conserved pathogen-associated molecular patters (PAMPs), such as lipopolysaccharide (LPS), peptidoglycan (PNG), lipoteichoic acid, and double-stranded viral RNA, can be discriminated by a wide range of pattern recognition receptors (PRRs) (Medzhitov and Janeway, 2000; Janeway and Medzhitov, 2002; Leulier and Lemaitre, 2008). An important class of PRRs is the Toll receptor superfamily, which includes vertebrate Toll-like receptors (TLRs) and invertebrate Tolls (Takeda and Akira, 2004; Hopkins and Sriskandan, 2005). When triggered by pathogens, the Toll pathway, such as in insects, is induced to activate Spätzle (Yuan et al., 2017). Afterward, Spätzle binds to Toll receptor and mediates downstream molecules containing Myeloid differentiation factor 88 (MyD88), Tube, Pelle, tumor necrosis factor receptor (TNFR)-associated factor 6 (TRAF6), Cactus, and Dorsal (Anderson, 2000; Silverman and Maniatis, 2001). These cascades ultimately activate NF-κB transcription factors, which regulate the synthesis of specific target genes, such as antibacterial peptide genes (AMPs) (Lemaitre and Hoffmann, 2007).

Toll receptor (dToll) is originally identified in *Drosophila melanogaster* and then considered as a core component of the anti-Gram-positive bacterial and anti-fungal response in *Drosophila* signaling pathway (Lemaitre et al., 1996; Kawai and Akira, 2006). At present, 9 Tolls (dToll1-9) in *Drosophila* (Bilak et al., 2003; Valanne et al., 2011) and 13 members of TLRs (TLR1-13) in mammals (Roach et al., 2005; Akira et al., 2006) have been identified. Tolls and their homolog TLRs are evolutionarily conserved and have crucial roles in the recognition of microbial pathogens (Beutler, 2004). They are a family of transmembrane proteins (type I protein) characterized by a transmembrane region, extracellular leucine-rich repeat (LRR) domains, and an intracellular Toll/interleukin-1 receptor (TIR) domain (Funami et al., 2004; Coscia et al., 2011). Upon the recognition of ligands by LRR domains, the cytoplasmic TIR domain interacts with downstream TIR-containing adaptor proteins, which activate the TLR-mediated signaling cascade (Akira et al., 2006; Valanne et al., 2011). A large number of Tolls/TLRs from various species are involved in the immune responses of hosts to infectious pathogens. For instance, a TLR (CfTLR) from Zhikong scallop *Chlamys farreri* can activate diverse downstream reactions, including antibacterial activity, antioxidant response, and apoptosis against *Vibrio anguillarum* (Wang et al., 2011). Four TLR homologs (CgTLRs) have been identified in *Crassostrea gigas*, and they have a consistent challenge response to multiple PAMPs and are involved in hemocyte activation and TNF induction induced by bacterial infection (Zhang et al., 2013). The expression of *CnTLR-1* increases after *Vibrio parahaemolyticus*, LPS, and Poly I:C challenge and acts as an important factor for an immune defense against pathogens in noble scallop (*Chlamys nobilis*) (Lu et al., 2016).

Triangle-shell pearl mussel (*Hyriopsis cumingii*) has been widely cultured for freshwater pearl production in China (Li et al., 2005). The frequent outbreak of diseases causes high mortality and large economic losses to mussel aquaculture. Understanding the native response against infectious diseases is crucial for the sustainable production of this species. In previous studies, seven Tolls, namely, *HcToll1* (Ren et al., 2013), *HcToll2-1* (Ren et al., 2014), *HcTollI*, *HcToll2-2* (Cao et al., 2016), *HcToll3* (Zhang et al., 2017), *HcToll4*, and *HcToll5* (Huang Y. et al., 2018), have been cloned and identified in *H. cumingii*, and they play crucial roles in bacterial and viral infections. However, the significance of mussel Tolls to immune responses at a protein level has been rarely discussed, and we have yet to fully elucidate the molecular mechanisms involved in how this pathway regulates immune responses. Here, two new Toll family members, designated as *HcToll6* and *HcToll7*, were identified in *H. cumingii*; their expression in tissues and changes after bacterial infection were examined. The functions of *HcToll6* and *HcToll7* were also analyzed in antibacterial immunity. The results of this study offer helpful information to characterize Tolls in mussels.

## Materials and Methods

### Experimental Animals

A total of 100 adult *H. cumingii* with a full shell length of 6–7 cm were obtained from a farm of Wuhu City, China. All the mussels were cultured in our laboratory aeration tank with recirculating water at 25°C for 2 weeks before any experiments.

### Immune Challenge and Tissues Collection

For the bacterial challenge, 50 μL of activated *Staphylococcus aureus* (3 × 10^7^ cells) or *V. parahaemolyticus* (3 × 10^7^ cells) were injected into the adductor muscles of *H. cumingii* (each group contained 20 mussels). Of these mussels, 20 were similarly injected with 1 mL of PBS (140 mM NaCl; 3 mM KCl; 8 mM Na_2_HPO_4_; 1.5 mM KH_2_PO_4_, pH 7.4) to serve as controls. The gills and the hepatopancreas of five mussels from each group were collected at 2, 6, 12, and 24 h post-injection and used for the isolation of total RNA. Tissues (hemocytes, hepatopancreas, gills, and mantle) were also collected from untreated mussels for tissue distribution studies.

### Double-Stranded RNA-Mediated RNAi Assay

The cDNA fragments amplified by four specific primers (HcToll6-RNAi-F: 5′-GCGTAATACGACTCACTATAGGCCCGGGCGTGGCAAGATG-3′ and HcToll6-RNAi-R: 5′-GCGTAATACGACTCACTATAGGCAGAGACTCGAGGTATGG-3′; HcToll7-RNAi-F: 5′-GCGTAATACGACTCACTATAGGGGAGAGAACTTGGACAGG-3′ and HcToll7-RNAi-R: 5′-GCGTAATACGACTCACTATAGGGGGCATTCAAAGGCAGACG-3′) linked to the T7 promoter were used as templates for the synthesis of double-stranded RNA (dsRNA) by using T7 RNA polymerase (Fermentas, Waltham, MA, United States) in accordance with previously described methods (Wang et al., 2012). The fragment of green fluorescent protein (GFP) was also obtained with the primers GFP-RNAi-F: 5′-GCGTAATACGACTCACTATAGGTGGTCCCAATTCTCGTGGAAC-3′ and GFP-RNAi-R: 5′-GCGTAATACGACTCACTATAGGCTTGAAGTTGACCTTGATGCC-3′ to synthesize the control dsRNA. Each mussel of the experimental groups (HcToll6-RNAi, HcToll7-RNAi) and the control group (GFP-RNAi) were coinjected with 15 μg of corresponding dsRNA and 50 μL of *V. parahaemolyticus* (3 × 10^7^ cells). At the same time, *V. parahaemolyticus* (3 × 10^7^ cells) alone was injected into the mussels as control. After 12 h post-injection, 15 μg of *dsHcToll6*, *dsHcToll7*, or *dsGFP* was injected into the same mussel. The gills of the mussel in each group were collected separately for RNA extraction 24 h after the last injection. The experiments were biologically repeated three times.

### RNA Extraction and First Strand cDNA Synthesis

Total RNA was extracted from the same tissues by using an RNApure high-purity total RNA rapid extraction kit (Spin-Column; BioTeke, Beijing, China) in accordance with the manufacturer’s protocol. The quality and concentration were checked through agarose gel electrophoresis and NanoDrop 2000 (Beijing GenoStar Biotech Co., Ltd., Beijing, China). First-strand cDNA synthesis for quantitative real-time PCR (qPCR) analysis was performed using a PrimeScript^®^ first-strand cDNA synthesis kit (Takara, Japan) to transcribe poly (A) mRNA with the Oligo-d(T) Primer and random 6-mer primers. The total RNA obtained from the hepatopancreas was also reverse transcribed via a Clontech SMARTer^TM^ RACE cDNA amplification kit (Clontech, Palo Alto, CA, United States) with 5′-CDS Primer A and SMARTerIIA oligos for 5′-RACE Ready cDNA, and 3′-CDS Primer A for 3′-RACE-Ready cDNA. Manufacturer-recommended reaction conditions were used.

### Cloning of *HcToll6* and *HcToll7* cDNAs

The partial cDNA sequences of *HcToll6* and *HcToll7* were retrieved from our previous hepatopancreas transcriptome database (unpublished). In this experiment, 3′ and 5′ RACE were performed by using a Clontech Advantage^®^ 2 PCR kit from Takara (Japan) with two pairs of specific primers (HcToll6-F:5′-TGGATAAACGATGTGCTAAGCGACCCC-3′, HcToll6-R: 5′-CTGTGAAGACCACGCAAATAGAGACGGAAC-3′; HcToll7-F: 5′-GAGAGAACTTGGACAGGAAAGGGGGCT-3′, HcToll7-R: 5′-CGTATCGGCAGGTCGCCAAGGGTAAC-3′) to obtain the full-length cDNAs of *HcToll6* and *HcToll7*. The amplification products were purified using a DNA gel extraction kit (Shanghai Generay Biotech Co., Ltd., Shanghai, China), inserted into the pEasy-T3 vector, and transformed into *Escherichia coli* Trans1-T1 cells (TransGen Biotech, Beijing, China). The putative clones were identified by PCR with M13F and M13R primers. The selected clones were sequenced by a commercial company (Springen, China).

### Bioinformatics Analysis

The BLAST algorithm^1^ was used to analyze the nucleotide and protein sequence homologs. The amino acid sequences of *HcToll6* and *HcToll7* were translated using ExPASy^2^. The signal peptide was predicted using SignalP 4.1 Server^3^, and the domain organization was identified by SMART^4^. The ExPASy Compute pI/Mw tool^5^ was used to predict the molecular weight and isoelectric point of HcToll6 and HcToll7. Multiple amino acid sequence alignments were generated with DNAMAN and GENDOC by using the ClustalX 2.0^6^. The phylogenetic relationships among the Toll homologs were determined by constructing a phylogenetic tree via the neighbor-joining (NJ)-embedded algorithm with MEGA 7 and the bootstrapping of 1000 replicates (Kumar et al., 2016).

### Quantitative Real-Time PCR Assay

qPCR was conducted in accordance with previously described methods (Huang et al., 2013) to examine the expression profiles of *HcToll6* and *HcToll7* transcripts in various samples. The gene specific primers for *HcToll6* (HcToll6-RT-F: 5′-AAGTGTTATCTGTTCCGTCTCT-3′ and HcToll6-RT-R: 5′-GTGTATGTTAGTGGGTGGTATCT-3′) and *HcToll7* (HcToll7-RT-F: 5′-AGCGAAGAAGAGAATGGG-3′ and HcToll7-RT-R: 5′-AAAGGCAGACGATAGATAGG-3′) were designed using Premier version 5.0. After *HcToll6* and *HcToll7* were knocked down, two immune-related gene expression levels were analyzed with the primers HcLyso-RT-F: 5′-CTTCTTTCTTGTTGGTCTGC-3′ and HcLyso-RT-R: 5′-CTGGTAGTAGCCACAGGACA-3′; HcDef-RT-F: 5′-GGTGTCGTCTATCTTGCTTC-3′ and HcDef-RT-R: 5′-AGGTTATTTGGTCATCTATTTTG-3′. β-actin was also amplified as a reference gene by using the primers β-actin-RT-F: 5′-GTGGCTACTCCTTCACAACC-3′ and β-actin-RT-R: 5′-GAAGCTAGGCTGGAACAAGG-3′. The data were calculated by the 2^-Δ ΔCt^ methods (Livak and Schmittgen, 2001) and were subjected to statistical analysis. An unpaired sample *t*-test was conducted, and differences were considered significant at *P* < 0.05 and extremely significant at *P* < 0.01.

### Recombinant Expression of LRRs of *HcToll6* and *HcToll7*

cDNA fragments encoding the LRR domains of *HcToll6* (480 amino acids) and *HcToll7* (446 amino acids) were amplified from the hepatopancreas cDNA by using the primers HcToll6-LRR-ex-F: 5′-GGATCCCCAGGAATTCCCACATGTCCAGCGAACTGCA-3′ and HcToll6-LRR-ex-R: 5′-GATGCGGCCGCTCGAGTTAGGTTGGTTTGGCATCTTTCAA-3′; HcToll7-LRR-ex-F: 5′-GGTGATCCCCAGGAATTCCCCCATCACTGATTCGATTTCT-3′ and HcToll7-LRR-ex-R: 5′-GATGCGGCCGCTCGAGTTATCTGCAATCTTCATCCGTTGG-3′. *Eco*RI and *Xho*I restriction sites were inserted at the beginning and end of the DNA fragments. After digestion occurred, the PCR products were cloned into the pGEX-6p-2 vector. Recombinant plasmids were transformed into *E. coli* BL21 (DE3) cells (TransGen Biotech, China), which were then cultured in Luria–Bertani (LB) medium (100 μg/mL ampicillin) at 37°C and 200 rpm. When OD600 of the culture reached 0.6, isopropyl-β-D-thiogalactoside (IPTG; 0.5 mM) was added. After 5 h of culture at 28°C and 200 rpm, the cells were collected by centrifugation at 6000 rpm for 10 min. They were then resuspended in PBS containing 0.1% Triton X-100. After cell sonication and centrifugation were accomplished, the proteins were purified using Glutathione Sepharose 4B chromatography (Gen-Script, United States) in accordance with the manufacturer’s instructions. The purified proteins were analyzed by 12.5% SDS-PAGE and stained with Coomassie brilliant blue G250.

### Microbial Binding Assay

Seven bacterial species, namely, *S. aureus*, *Micrococcus luteus*, *Bacillus subtilis*, *Bacillus thuringiensis*, *V. parahaemolyticus*, *Aeromonas hydrophila*, and *E. coli*, were used to measure the binding activities of rHcToll6-LRR and rHcToll7-LRR by using a binding assay described previously (Huang et al., 2014). After the bacteria were cultivated, the microorganisms were collected by centrifugation at 6000 rpm for 5 min. Microbial pellets were washed three times with tris buffered saline (TBS; 20 mM Tris–HCl, 150 mM NaCl, pH 7.4) and resuspended in TBS. Each microorganism (approximately 2 × 10^8^ cells) was incubated in 100 μg of rHcToll6-LRR or rHcToll7-LRR by gentle rotation for 60 min at 37°C. After centrifugation at 6000 rpm for 5 min, the harvested cells were rinsed three times with TBS. All of the fractions were analyzed through 12.5% SDS-PAGE, transferred onto a nitrocellulose membrane, and revealed through Western blot analysis by using an anti-GST rabbit antibody (TransGen, Beijing, China).

### Pathogen-Associated Molecular Patter Binding Assay

Enzyme-linked immunosorbent assay (ELISA) was conducted on the basis of a previous study (Huang et al., 2014) to detect the direct binding of rHcToll6-LRR and rHcToll7-LRR to LPS (*E. coli* serotype 055: B5) and PGN (*M. luteus*) (Sigma, St. Louis, MO, United States). LPS and PGN were initially dissolved in sterile water at a concentration of 80 μg/mL. Each well of the 96-well plate was then coated with 50 μL of sugar solution. The plate was then incubated overnight at 37°C and heated for 30 min at 60°C. Each well was blocked with 200 μL of bovine serum albumin (BSA; 1 mg/mL) at 37°C for 2 h and then washed with TBS four times. The purified proteins with gradient dilution (0.78, 1.56, 3.125, 6.25, 12.5, and 25 μg/mL in TBS containing 0.1 mg/mL BSA) were added to the wells and incubated at 37°C for 3 h. The wells were washed four times. Afterward, 100 μL of rabbit monoclonal anti-GST antibody (1/2000 diluted in 0.1 mg/mL BSA) was then added and incubated at 37°C for 1 h. Each well was washed four times and then incubated with 100 μL of peroxidase-conjugated goat anti-rabbit IgG (1/5000 diluted) at 37°C for 1 h. The plate was washed four times with TBS and developed with 0.01% 3,3′,5,5′-tetramethylbenzidine (Sigma). Approximately 2 M H_2_SO_4_ was used to stop the reaction. Absorbance was read and noted at 450 nm by using a plate reader (BioTek Instruments, Winooski, VT, United States). The assays were performed in triplicate.

### Bacteria Inhibition Assay

The inhibitory effects of rHcToll6-LRR and rHcToll7-LRR on *S. aureus* and *V. parahaemolyticus* were tested as described in our previous report with slight modifications (Huang Y. et al., 2016). In this experiment, 50 μL of bacteria was transferred to 5 mL of LB broth, and rHcToll6-LRR and rHcToll7-LRR were separately added at a final concentration of 100 μg/mL. TBS and GST-tag protein served as a blank group and a negative control group, respectively. Each sample was incubated at 37°C with shaking at 200 rpm, and bacterial growth was monitored by checking OD600 every 1 h.

## Results

### Molecular Cloning and Sequence Characterization of *HcToll6* and *HcToll7*

**FIGURE 1:**
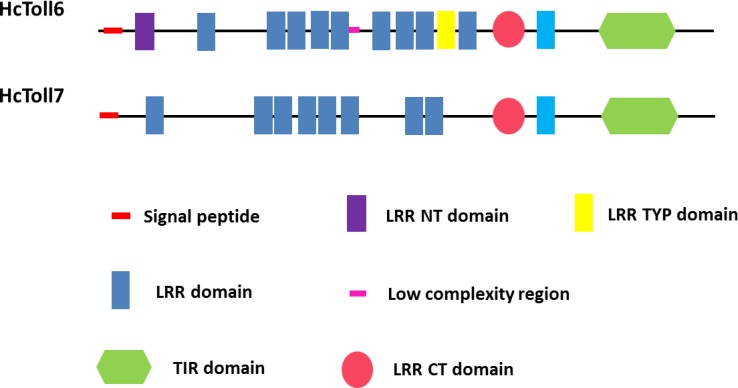
Domain organizations of *HcToll6* and *HcToll7* from *H. cumingii*.

The complete cDNA sequences of *HcToll6* and *HcToll7* were obtained by using RACE technology. The full-length *HcToll6* cDNA was 3207 bp, and it consisted of a 2223 bp open reading frame (ORF) that encoded a peptide of 740 amino acids, a 52 bp 5′ untranslated region (UTR), and a 932 bp 3′ UTR with a potential polyadenylation signal (AATAAA) and a poly (A) tail (Supplementary Figure S1A). SignalP analysis showed that HcToll6 had a signal peptide with 24 amino acids (aa) at the N-terminal. SMART analysis predicted that the deduced HcToll6 protein contained 12 LRR residues, including nine 21–30 aa LRRs, a 32 aa LRR N-terminal domain (LRR-NT), a 24 aa LRR typical (most populated) subfamily domain (LRR-TYP), and a 55 aa LRR C-terminal domain (LRR-CT) in the extracellular domain, a 23 aa transmembrane domain, and a 141 aa intracellular TIR domain (Figure 1). The molecular mass of the mature protein was 86.1 kDa, and the estimated isoelectric point was 7.04. *HcToll7* cDNA was 3216 bp, and it was composed of an 84 bp 5′ UTR, a 993 bp 3′ UTR with poly (A), and a 2139 bp ORF encoding a 712 aa HcToll7 protein with a deduced molecular mass of 82.1 kDa and a theoretical pI of 8.69 (Supplementary Figure S1B). Similar to *HcToll6* domain organization, *HcToll7* contains a 31 aa signal peptide, eight 20–24 aa LRR domains, a 58 aa LRR-CT domain, a 20 aa transmembrane domain, and a 142 aa TIR domain (Figure 1).

### Homologous and Phylogenetic Analysis

**FIGURE 2:**
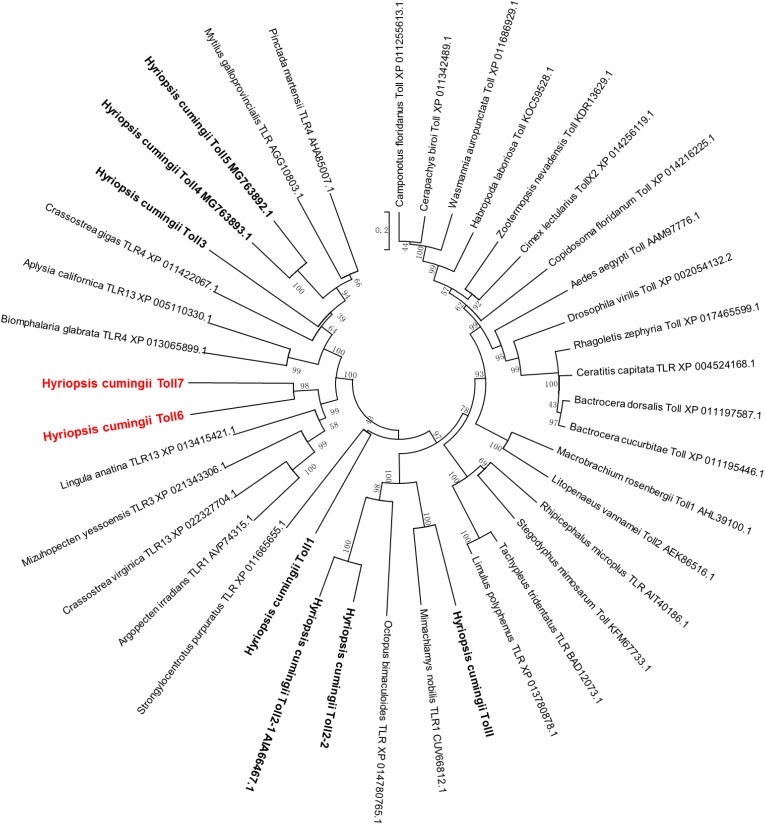
Phylogenetic analysis of *HcToll6* and *HcToll7* with other Tolls or TLRs. The repeatability of the results was checked by performing 1000 bootstraps on the NJ trees. The scale bar indicates a branch length of 0.2.

The multiple sequence alignment analysis of *HcToll6* and *HcToll7* showed that they had different amino acid compositions (Supplementary Figure S2). BLAST analysis indicated that the deduced amino acid sequences of *HcToll6* and *HcToll7* were similar to the amino acid sequences of the Tolls from *Mytilus galloprovincialis* (XP_021343306.1, 28, 31% similarity), *Crassostrea virginica* (XP_022327704.1, 28, 31% similarity), *Argopecten irradians* (AVP74315.1, 26, 31% similarity), and *Lingula anatina* (XP_013415421.1, 25, 28% similarity). A phylogenetic tree was constructed on the basis of the homologous analysis of 9 Tolls from *H. cumingii* and 31 Tolls from other species. The relationship showed that the Tolls of *H. cumingii* were clustered independently, and *HcToll6* and *HcToll7* had a relatively close evolutionary position with the reported Tolls from *M. galloprovincialis*, *C. virginica*, *A. irradians*, and *L. anatina* (Figure 2).

### Tissue Distributions of *HcToll6* and *HcToll7*

**FIGURE 3:**
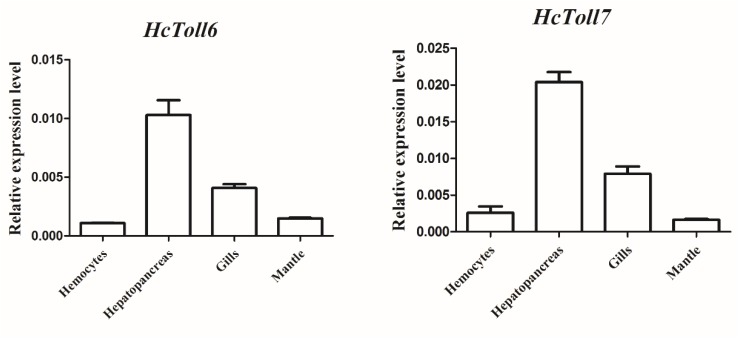
Expression profiles of *HcToll6* and *HcToll7* in various tissues. The 2^-Δ ΔCt^ method was utilized in the analysis of the expression patterns of *HcToll6* and *HcToll7* with β-actin as the reference gene. Error bars represent SD.

The tissue distributions of *HcToll6* and *HcToll7* were investigated through qPCR. *HcToll6* and *HcToll7* transcriptions were constitutively expressed in the tested tissues, including hemocytes, hepatopancreas, gills, and mantle, when their expression levels were normalized to the housekeeping gene β-actin. *HcToll6* and *HcToll7* mRNAs were highly expressed in the hepatopancreas, moderately expressed in the gills, and weakly expressed in the hemocytes and the mantle (Figure 3).

### Transcriptional Regulation of *HcToll6* and *HcToll7* in Response to *S. aureus* and *V. parahaemolyticus* Stimulation

**FIGURE 4:**
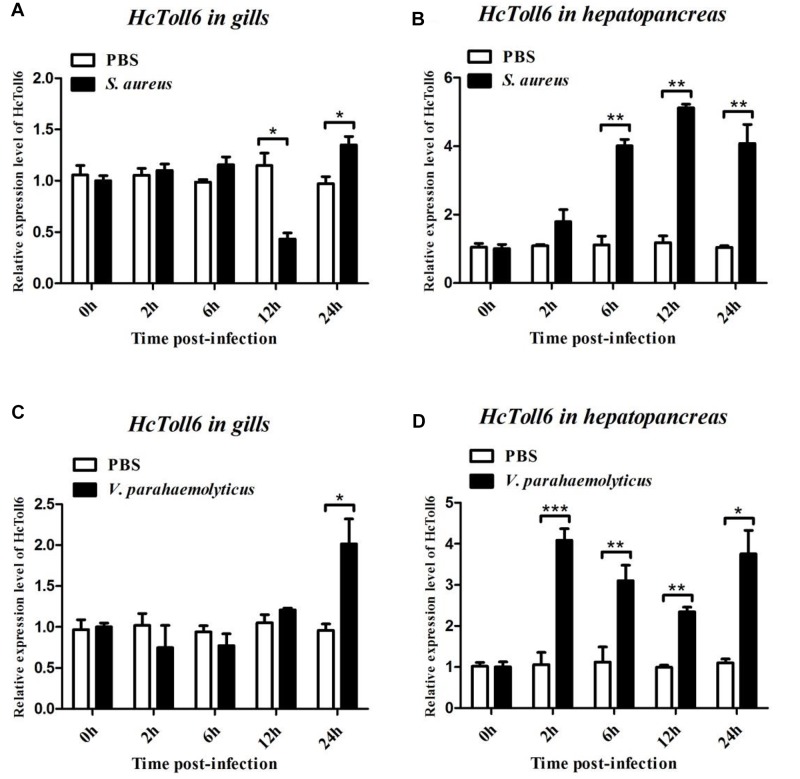
Time-course expression analysis of *HcToll6* gene in the gills **(A,C)** and hepatopancreas **(B,D)** at 0, 2, 6, 12, and 24 h after injection with *S. aureus* and *V. parahaemolyticus*. Expression level was determined by qPCR and normalized to β-actin in triplicate. Statistically significant differences were analyzed by *t*-test and indicated by asterisks (^∗^*P* < 0.05, ^∗∗^*P* < 0.01, and ^∗∗∗^*P* < 0.001).

**FIGURE 5:**
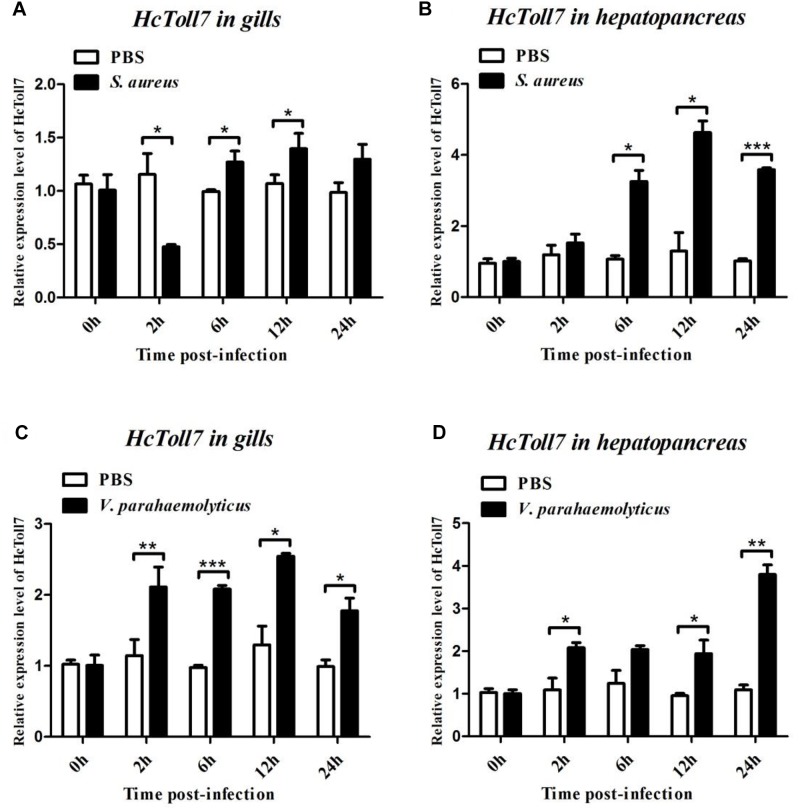
Time-course expression analysis of *HcToll7* gene in the gills **(A,C)** and hepatopancreas **(B,D)** at 0, 2, 6, 12, and 24 h after injection with *S. aureus* and *V. parahaemolyticus*. Expression level was determined by qPCR and normalized to β-actin in triplicate. Statistically significant differences were analyzed by *t*-test and indicated by asterisks (^∗^*P* < 0.05, ^∗∗^*P* < 0.01, and ^∗∗∗^*P* < 0.001).

After treatment with *S. aureus*, the *HcToll6* transcript level in the gills was obviously downregulated at 12 h and then gradually upregulated at 24 h (Figure 4A), whereas *HcToll7* significantly decreased at 2 h and kept a relatively high level from 6 h to 24 h (Figure 5A). *HcToll6* and *HcToll7* transcription levels in the hepatopancreas were upregulated gradually from 2 h, reached a peak at 12 h, and subsequently remained relatively high until 24 h (Figure 4B, 5B). After the *V. parahaemolyticus* challenge, the mRNA expression of *HcToll6* in the gills slowly decreased at 2 h, gradually increased, and reached a maximum level at 24 h (Figure 4C). *HcToll7* expression increased at 2 h, peaked at 12 h, and decreased at 24 h (Figure 5C). In the hepatopancreas, *HcToll6* expression reached its first peak at 2 h and decreased from 6 h to 12 h. The expression level reached the second peak at 24 h (Figure 4D). *HcToll7* expression increased continuously from 2 h to 12 h and peaked at 24 h (Figure 5D). No changes in the *HcToll6* and *HcToll7* expression levels of the PBS-injected group were detected for 24 h.

### Effect of the *HcToll6* and *HcToll7* Knockdown on the Expression of AMPs

**FIGURE 6:**
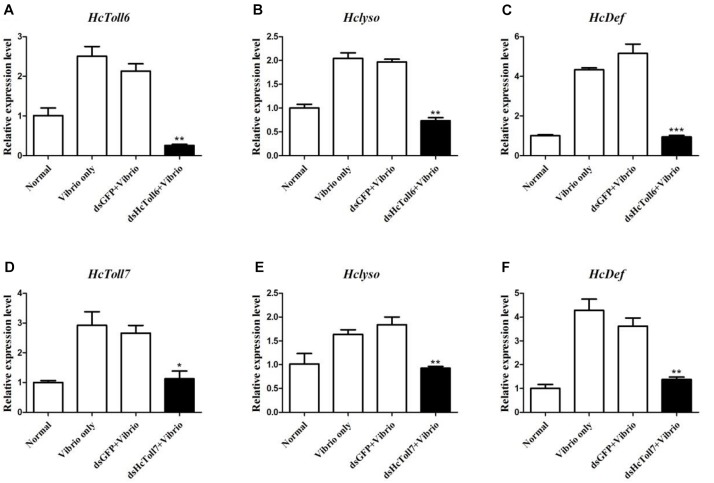
AMP expression patterns in *HcToll6* **(A–C)** and *HcToll7* **(D–F)** knockdown *H. cumingii* after *V. parahaemolyticus* infection. The mussels were divided into four groups (Normal, *V. parahaemolyticus* only, *dsGFP* and *V. parahaemolyticus*, *dsHcToll6*/*dsHcToll7*, and *V. parahaemolyticus*). The expression profiles of AMPs (*HcLyso* and *HcDef*) in the gills at 36 h after the first injection was investigated by qPCR. All data were obtained from at least three parallel experiments and were expressed as mean ± SD values; asterisks indicate significant differences (^∗^*P* < 0.05, ^∗∗^*P* < 0.01, and ^∗∗∗^*P* < 0.001).

*HcToll6* and *HcToll7* in *H. cumingii* were knockdown by *dsHcToll6* and *dsHcToll7*, respectively, to investigate the contribution of *HcToll6* and *HcToll7* in mussel immunity. In Figure 6A,D, the transcriptional levels of *HcToll6* and *HcToll7* in the gills were suppressed after *dsHcToll6* and *dsHcToll7* injection relative to those of the normal group, indicating that silencing was gene specific. The mRNA expression levels of lysozyme (*HcLyso*) and defensin (*HcDef*) in the gills were highly induced after stimulation with *V. parahaemolyticus*. The transcripts of these genes were considerably downregulated in the gills of *HcToll6*-silenced and *HcToll7*-silenced mussels challenged with live bacteria in comparison with those in the *dsGFP* and *V. parahaemolyticus* only injection groups (Figure 6B,C,E,F). These results implied that *HcToll6* and *HcToll7* might play a positive role in the regulation of AMPs.

### Expression and Purification of Recombinant HcToll6-LRR and HcToll7-LRR

**FIGURE 7:**
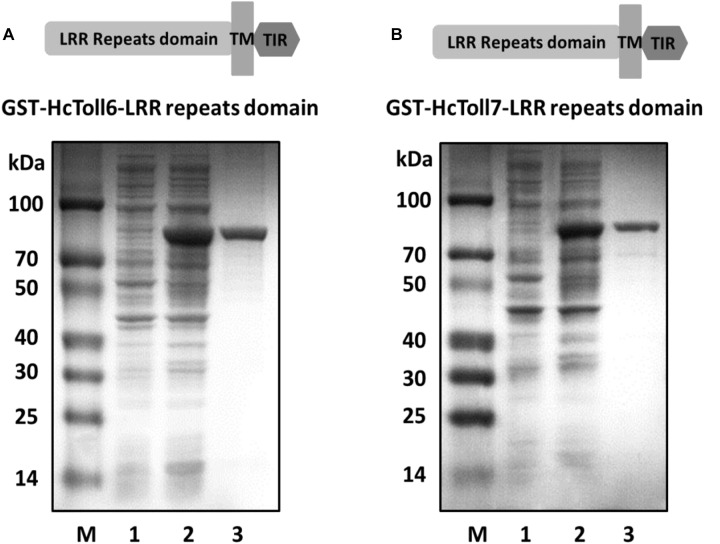
Pattern of recombinant HcToll6-LRR **(A)** and HcToll7-LRR **(B)** analyzed by 12.5% SDS-PAGE. Lane M, molecular weight markers; lane 1, total proteins of plasmids without induction; lane 2, total proteins of plasmids with IPTG induction; lane 3, purified rHcToll6-LRR/rHcToll7-LRR.

The recombinant plasmids of pGEX-6p-2-HcToll6-LRR and pGEX-6p-2-HcToll7-LRR were expressed in the prokaryotic expression system after transformation into *E. coli* BL21 (DE3). After IPTG induction, SDS-PAGE analysis revealed that two proteins with approximate molecular weights of 81.7 and 77.7 kDa were induced (Figure 7A,B, lanes 2), which exactly matched the predicted molecular mass deduced from *HcToll6* and *HcToll7*. Two purified products after Glutathione Sepharose 4B chromatography purification had only one band of approximately 81.7 and 77.7 kDa, which were the same size as the expected recombinant HcToll6-LRR (55.7 kDa) and HcToll7-LRR (51.7 kDa) with a 26 kDa vector GST-tag fragment (Figure 7A,B, lanes 3).

### Direct Binding of rHcToll6-LRR and rHcToll7-LRR to Microorganisms and Polysaccharides

**FIGURE 8:**
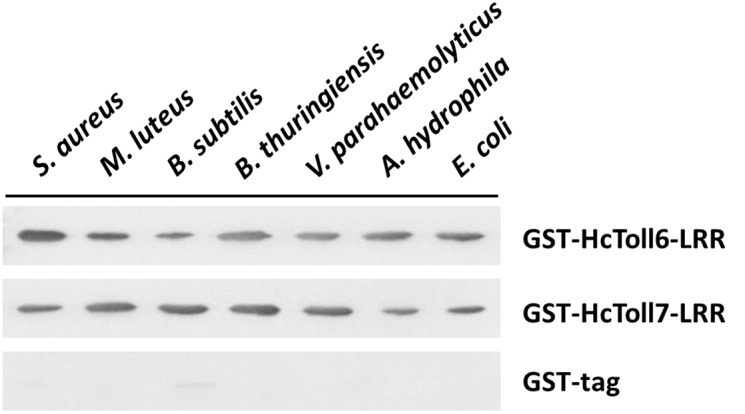
Binding of rHcToll6-LRR and rHcToll7-LRR toward microorganisms (Gram-positive bacteria *S. aureus*, *M. luteus*, *B. subtilis*, *B. thuringiensis* and Gram-negative bacteria *V. parahaemolyticus*, *A. hydrophila*, *E. coli*) were detected by Western blot analysis.

**FIGURE 9:**
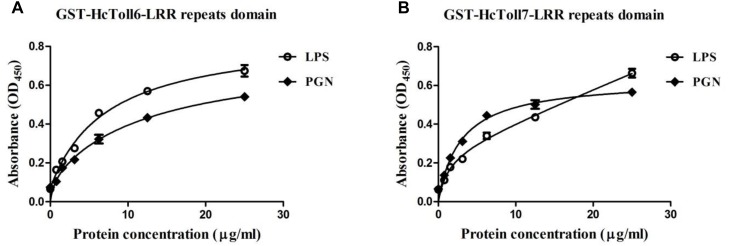
Binding of rHcToll6-LRR **(A)** and rHcToll7-LRR **(B)** to polysaccharides determined by ELISA. ELISA was used to quantify the binding of purified rHcToll6-LRR/rHcToll7-LRR to immobilized LPS and PGN.

An *in vitro* binding assay was conducted to test the binding of rHcToll6-LRR and rHcToll7-LRR to microorganisms. Purified rHcToll6-LRR and rHcToll7-LRR could bind to Gram-positive bacteria (*S. aureus*, *M. luteus*, *B. subtilis*, and *B. thuringiensis*) and Gram-negative bacteria (*V. parahaemolyticus*, *A. hydrophila*, and *E. coli*) at different band intensities. Figure 8 shows that rHcToll6-LRR could bind more tightly to *S. aureus* than to others. With the same protein contents, rHcToll7-LRR seemed to bind to most tested bacteria more potently than rHcToll6-LRR (Figure 8). In Figure 9, the direct binding specificity of rHcToll6-LRR and rHcToll7-LRR to sugars was tested through ELISA. Purified rHcToll6-LRR and rHcToll7-LRR could bind to LPS and PGN in a dose-dependent manner. The binding activity of rHcToll6-LRR to LPS was higher than that of PGN, whereas rHcToll7-LRR exhibited a similar binding affinity to LPS and PGN. They could bind to a broad spectrum of microorganisms and polysaccharides, although they had distinct specificities.

### Antimicrobial Activity Assay for rHcToll6-LRR and rHcToll7-LRR

**FIGURE 10:**
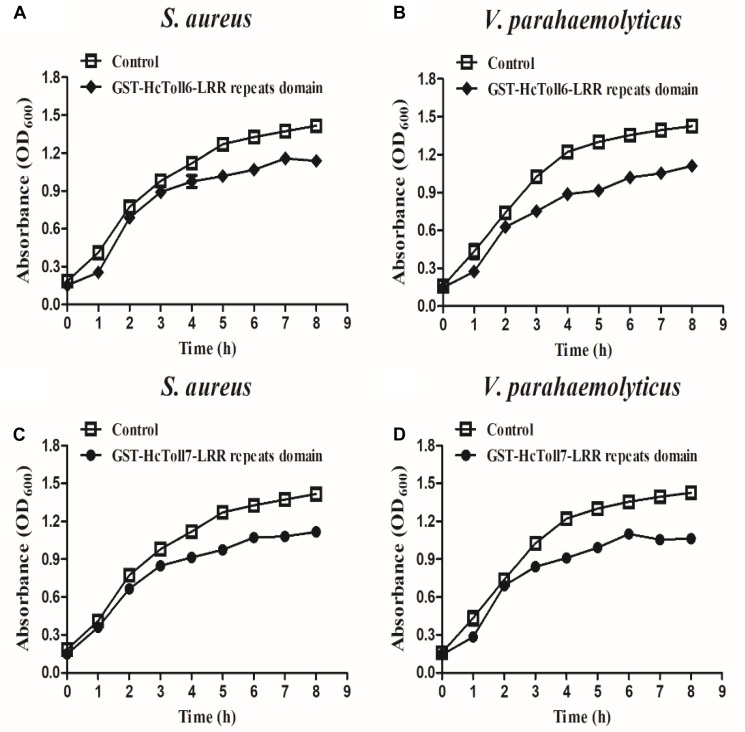
Bacterial growth inhibitory activities of rHcToll6-LRR and rHcToll7-LRR. Antimicrobial activities against cultured *S. aureus* **(A,B)** and *V. parahaemolyticus* **(C,D)** are shown by the inhibition growth curves. Bacteria were mixed with 100 μg/mL purified rHcToll6-LRR/rHcToll7-LRR (TBS as the control), and OD600 values were measured every 1 h. Data represent mean ± SD values from three independent replications. Error bars represent SD.

rHcToll6-LRR and rHcToll7-LRR (100 μg/mL; TBS as the control) were used to examine their inhibitory effects on the growth of *S. aureus* and *V. parahaemolyticus* at different times and to determine the antimicrobial activity. The results showed that the growth of *S. aureus* and *V. parahaemolyticus* incubated with rHcToll6-LRR and rHcToll7-LRR for 8 h was inhibited significantly compared with that of the control group (Figure 10). No obvious inhibitory activity was observed against the bacteria with GST-tag protein(data were not shown).

## Discussion

Tolls/TLRs play crucial roles in innate immunity by detecting invading microbes and thereby relaying downstream signaling cascades to activate the synthesis of AMPs and eliminate pathogens (Medzhitov, 2001). Recently, a large number of Tolls have been found in mollusks and confirmed to be involved in a range of innate immune responses. Our study was an attempt to elucidate the effect of Tolls in the immune response of mussels to bacterial infections.

In the present study, two Tolls, possessing 2223 and 2193 bp ORFs, were identified in the triangle sail mussel *H. cumingii* and named *HcToll6* and *HcToll7*. The deduced *HcToll6* and *HcToll7* proteins share two common structures: extracellular LRR domains and intracellular TIR domain. In contrast to the highly conserved TIR domain, the LRR domains show distinct divergence in sequence and are characterized by tandem copies of LRR motifs (Matsushima et al., 2007). BLAST and multiple sequence alignment analysis of proteins indicated that these two amino acid sequences were not conserved. Moreover, in the phylogenetic tree, HcToll6 and HcToll7 were closely matched to bivalve Tolls (MgToll, CvToll, AiToll, and LaToll) but were not clustered with other known *H. cumingii* Tolls. These findings indicated the novelty of HcToll6 and HcToll7 in *H. cumingii*.

In mussels, *HcToll6* and *HcToll7* were predominantly expressed in immune tissues, such as the hepatopancreas and gills. Gills are water-air-exchange tissues that are frequently exposed to external environments and in direct contact with pathogens (Huang X. et al., 2016). Similar to the fat body of insects and liver of mammals, the hepatopancreas is vital for the immune system and homeostasis maintenance (Jiravanichpaisal et al., 2006). A diverse set of tissues expressing *HcToll6* and *HcToll7* implies that these genes are biologically important. The mussels were infected with *S. aureus* and *V. parahaemolyticus* to explore the expression of *HcToll6* and *HcToll7* after bacteria challenge. qPCR confirmed that *HcToll6* and *HcToll7* mRNAs in hepatopancreas and gills were remarkably upregulated when stimulated with bacteria. This result is consistent with many previous reports (Ren et al., 2013; Zhang et al., 2017; Huang Y. et al., 2018). After treatment with different bacteria, the transcript levels of *HcToll6* and *HcToll7* in different tissues obviously varied. This finding indicated that the Toll functions are diverse, and that Toll exerts different immune effects in response to different stimuli. In invertebrates, the innate immune system recognizes Gram-positive bacteria and fungi mainly through the Toll signaling pathway, and the identification of Gram-negative bacteria mainly depends on the IMD pathway (Parker et al., 2001). In our study, Gram-positive and Gram-negative bacteria could regulate *HcToll6* and *HcToll7* expression and activate the Toll pathway. This result indicated that different immune regulatory mechanisms might be linked.

Toll was originally identified as an essential molecule for embryonic dorsal/ventral patterning in *Drosophila* and confirmed to be responsible for the modulation of a large number of AMPs (Valanne et al., 2011). *In vitro* dsRNA was synthesized and used to silence the expression of *HcToll6* and *HcToll7* and to examine the role of *HcToll6* and *HcToll7* in mediating the expression of AMPs. RNA interference (RNAi) involving dsRNA to silence genes has been applied to studies on mussel immunity (Huang D. et al., 2018; Zhao et al., 2018). The successful knockdown of *HcToll6*/*HcToll7* with a unique *dsHcToll6*/*dsHcToll7* provided a practical way of demonstrating their potential function in the innate immune system of mussels. The data showed that *HcToll6* and *HcToll7* RNAi strongly decreased the expression of *HcLyso* and *HcDef* in the gills of RNAi-treated mussels with *V. parahaemolyticus* challenge and the GFP RNAi group showed no change in gene expression compared with *V. parahaemolyticus* only group. Lysozymes are the first-line defensive enzymes to resist various invading pathogens and play key roles in the natural defense of most living organisms (D€uring et al., 1999). Defensins are AMPs that serve as a component of the immune defense system of many organisms and possess remarkable microbicidal activities (Wang et al., 2014). We speculated that *HcToll6* and *HcToll7* were involved in *H. cumingii* immune response challenged with *V. parahaemolyticus* and was related to the activation of *HcLyso* and *HcDef* expression. Nonetheless, the exact mechanism is unclear and needs an in-depth research.

Next, we found that the prokaryotic expressed recombinant LRR domains of HcToll6 and HcToll7 (rHcToll6-LRR and rHcToll7-LRR) have the ability to binding a wide range of four Gram-positive bacteria and three Gram-negative bacteria. To further explore the possible recognition mechanism, two common PAMPs were selected to detect the binding affinity of rHcToll6-LRR and rHcToll7-LRR, which showed similar affinity to LPS and PGN. The binding property of rHcToll6-LRR and rHcToll7-LRR were quite different from TLRs in mammals and *Drosophila* but was similar to those of rCgTLR6 or rMjToll1-3, which also had a wide spectrum of binding activities towards various bacteria and LPS and PGN (Wang et al., 2016; Sun et al., 2017). In view of the lack of adaptive immunity, invertebrates were suspected to harbor more PRRs by gene expansion as a compensation to identify diverse pathogens (Wang et al., 2016). More interestingly, rHcToll6-LRR and rHcToll7-LRR exhibited significant bactericidal activity against *V. parahaemolyticus* and *S. aureus* *in vitro*, which confirmed the significant role of Tolls in eliminating invading non-self. Based on these results, we draw a conclusion that HcToll6 and HcToll7 could play a primary role in anti-bacterial defense of *H. cumingii*. Our study provides more evidence and novel understanding to the potential functions of Tolls in the innate immunity of mussels.

## Ethics Statement

We declare that appropriate ethical approval and licenses were obtained during our research.

## Author Contributions

YH carried out the experiments and contributed reagents and materials. YH, GSZ, and QR designed the experiments, analyzed the data, and wrote the manuscript. All authors gave final approval for publication.

## Conflict of Interest Statement

The authors declare that the research was conducted in the absence of any commercial or financial relationships that could be construed as a potential conflict of interest.

## Footnotes

1 https://blast.ncbi.nlm.nih.gov/Blast.cgi

2 http://web.expasy.org/

3 http://www.cbs.dtu.dk/services/SignalP/

4 http://smart.embl-heidelberg.de/

5 http://web.expasy.org/compute_pi/

6 http://www.ebi.ac.uk/tools/clustalw2
