# Supplementary material for: Molecular Characterization of Two Toll Receptors in Hyriopsis cumingii and Their Potential Roles in Antibacterial Response
Source: Front Physiol. 2019 Jul 25;10:952. doi: 10.3389/fphys.2019.00952 (PMC6672746; doi:10.3389/fphys.2019.00952)
Supplement: Supplementary file 1 [file Data_Sheet_1.docx]

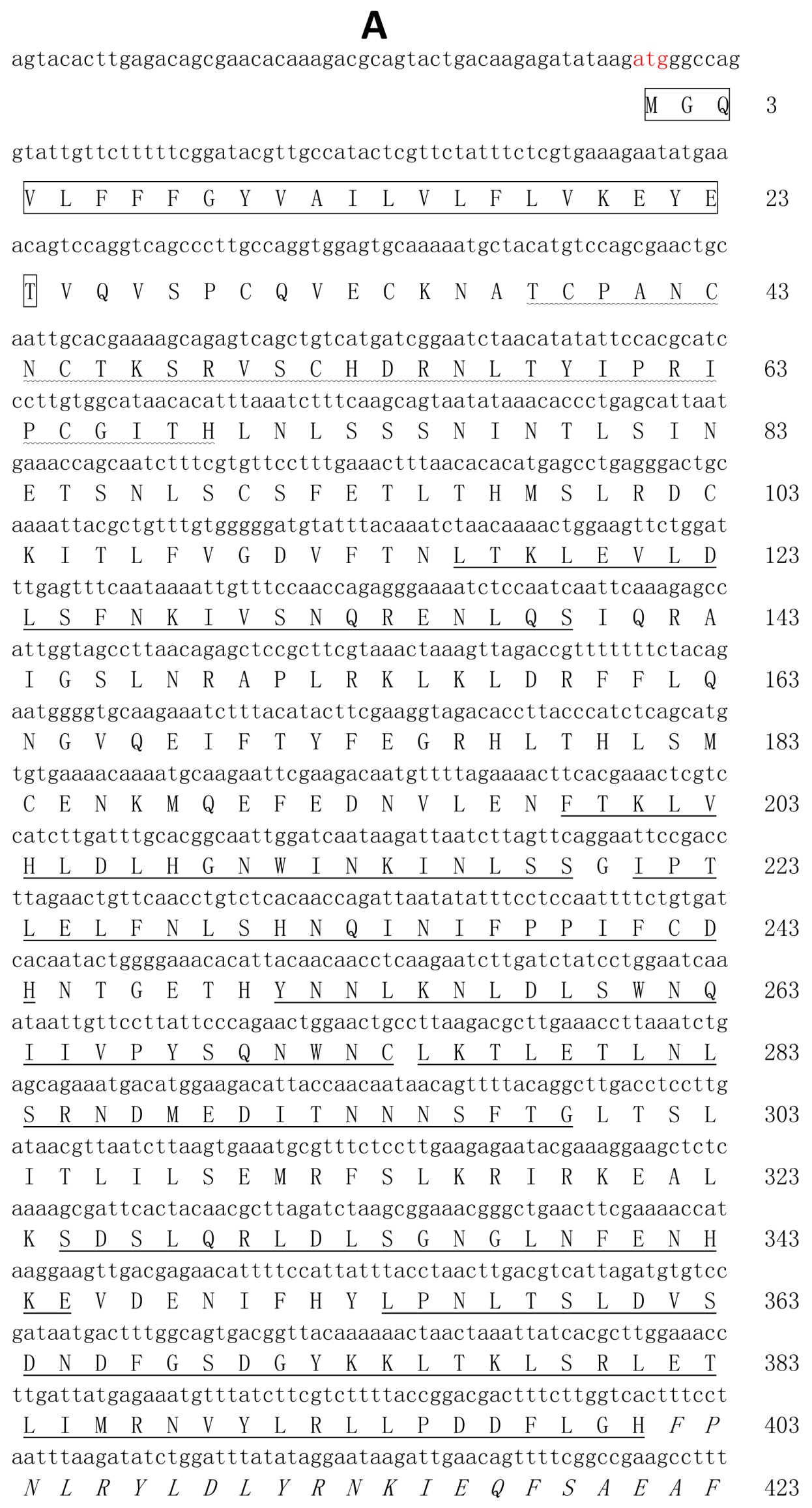


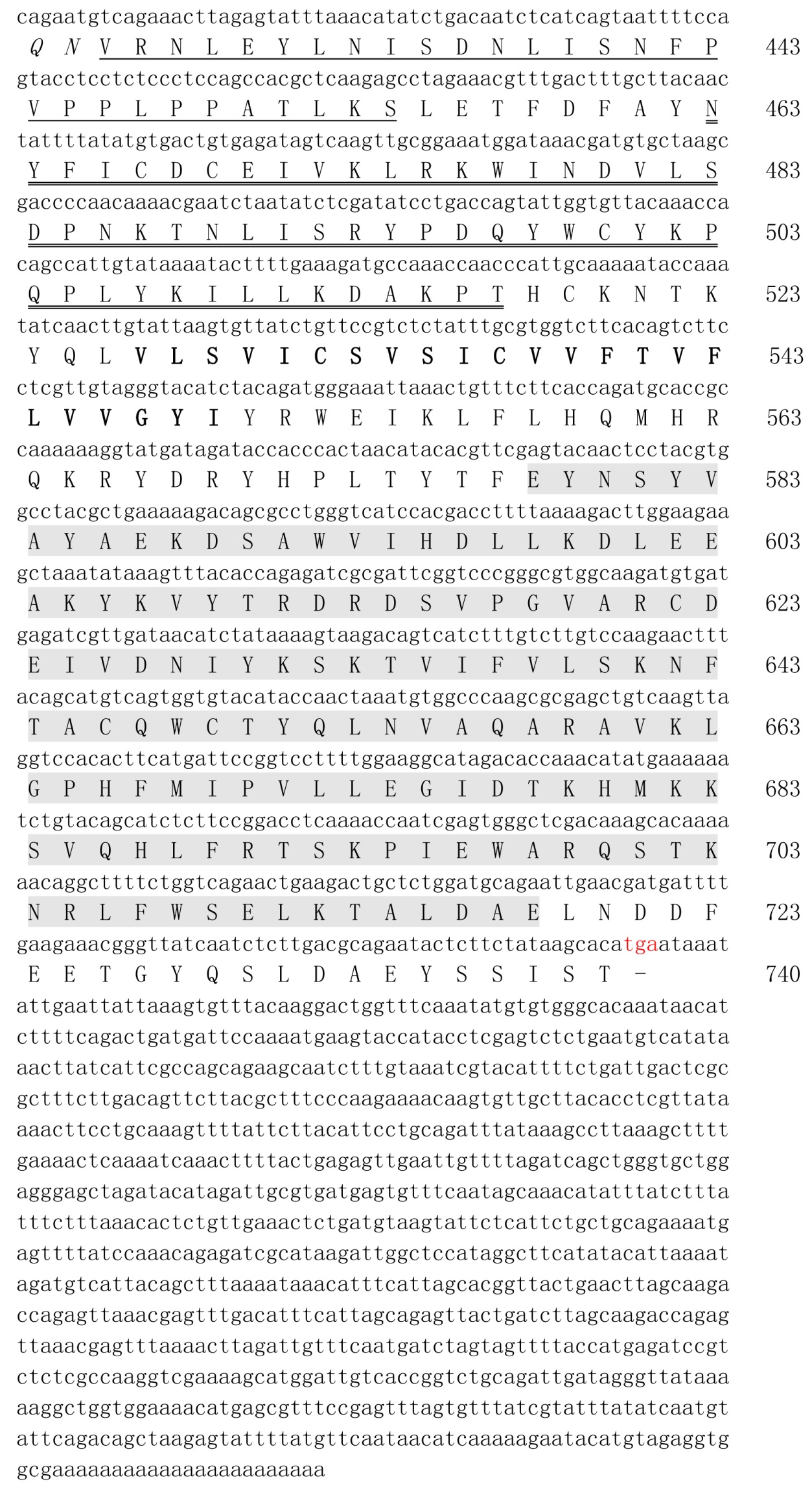


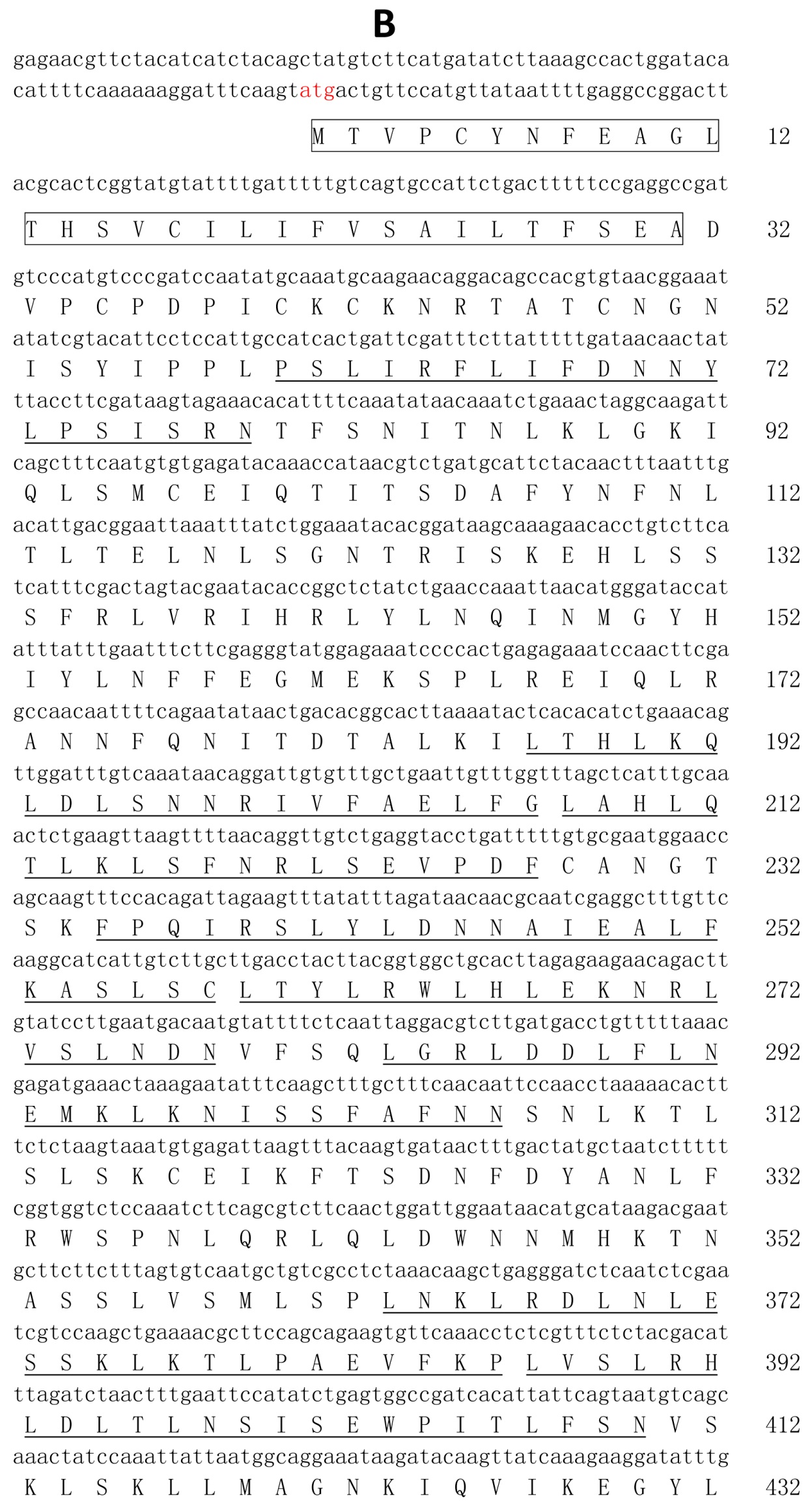


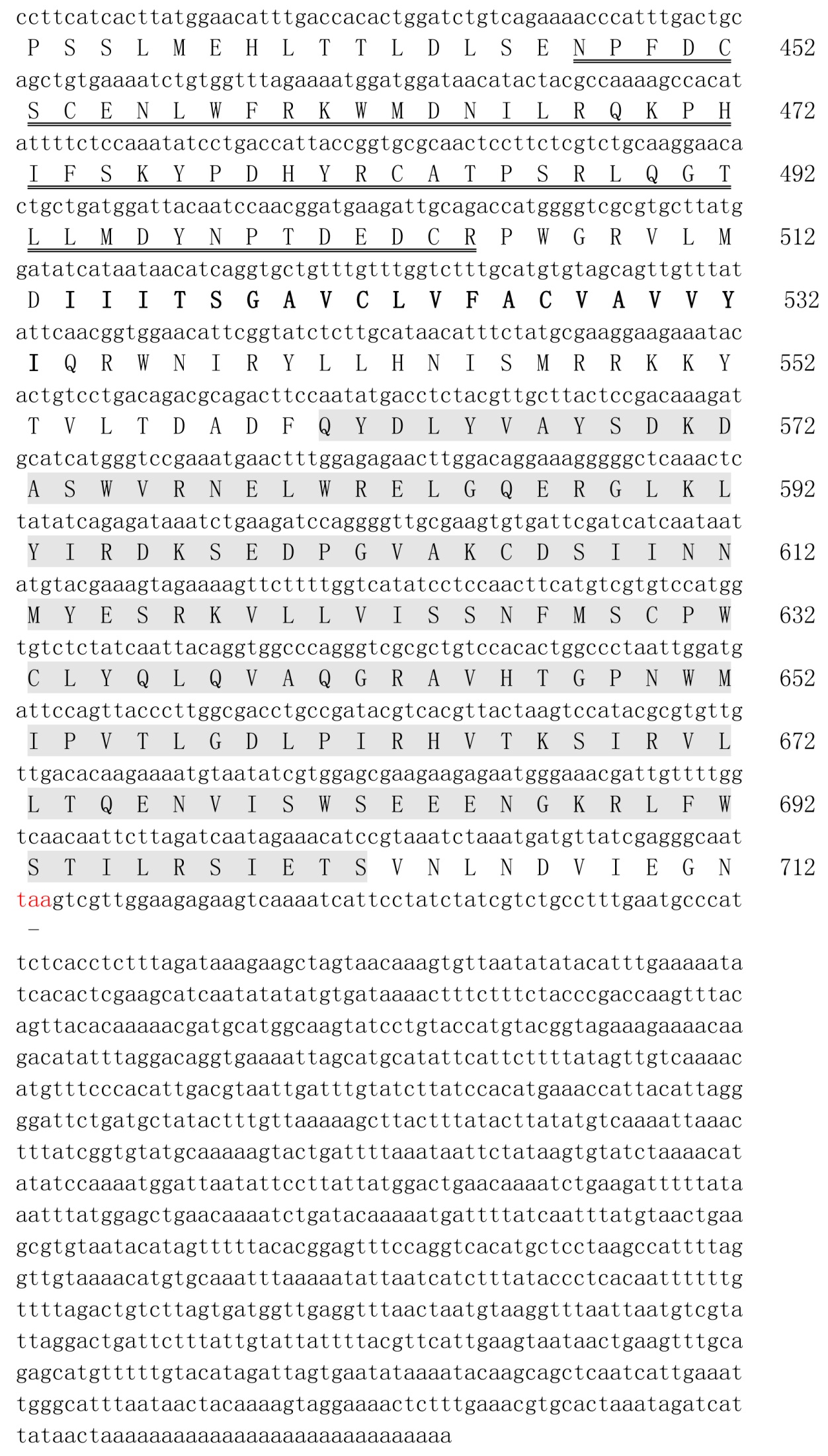


**FIGURE S1** Nucleotides and deduced amino acid sequences of HcToll6 **(A)** and HcToll7 **(B)** from *H. cumingii*. Signal peptides are boxed. The wavy lines represent the LRR NT domains. The LRR domains are underlined, the LRR TYP domains are labeled in italics, and the LRR CT regions are double underlined. Transmembrane domains are shown in bold, and shaded sequences denote the TIR domains.


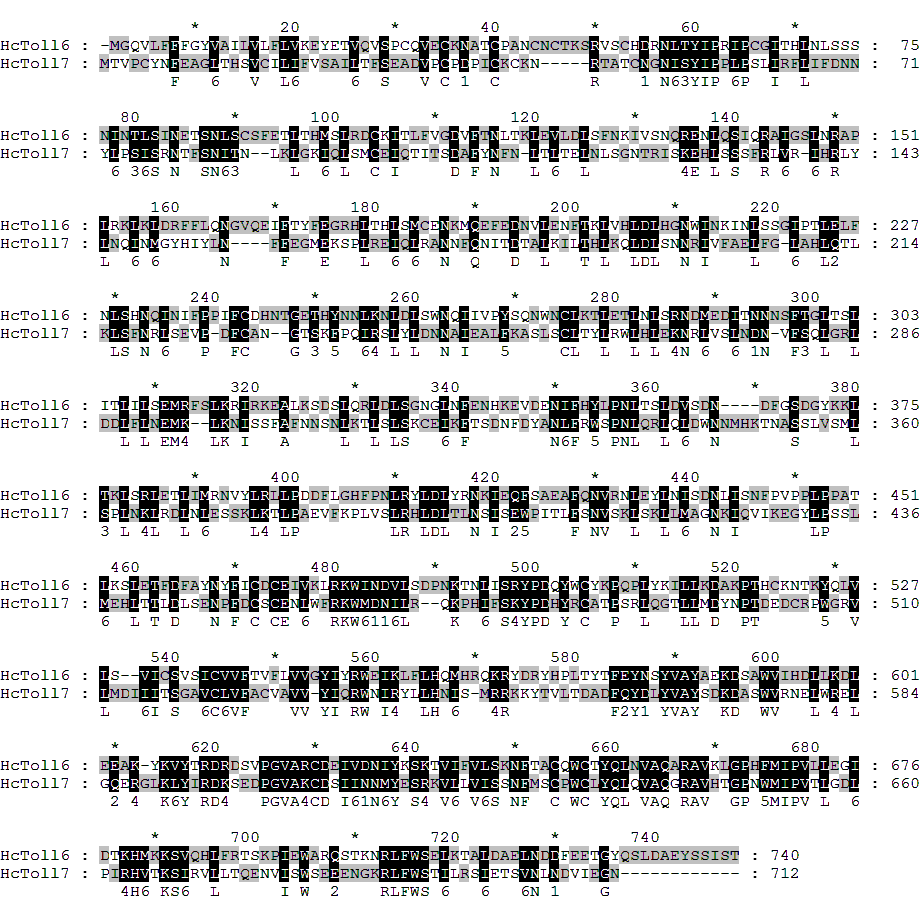


**FIGURE S2** Multiple sequence alignment by DNAMAN and GENDOC of HcToll6 and HcToll7 from *H. cumingii*.
